# Incidence of Newly Diagnosed Tuberculosis among Healthcare Workers in a Teaching Hospital, Thailand

**DOI:** 10.29024/aogh.2304

**Published:** 2018-08-31

**Authors:** Ploy Pongwittayapanu, Thunyarat Anothaisintawee, Kumthorn Malathum, Chathaya Wongrathanandha

**Affiliations:** 1 Department of Family Medicine, Faculty of Medicine Ramathibodi Hospital, Mahidol University, TH; 2 Department of Medicine, Faculty of Medicine Ramathibodi Hospital, Mahidol University, TH

## Abstract

**Background:**

Data on the incidence of new onset tuberculosis (TB) infection among healthcare workers (HCWs) in Thailand was scarce and not current.

**Objectives:**

To determine the incidence of TB, as well as the impact of TB on HCWs in a teaching hospital in Bangkok, Thailand.

**Methods:**

A time series cross-sectional study was conducted at Ramathibodi Hospital, Bangkok, Thailand. It was a teaching hospital with 9,562 employees. Medical records of personnel with TB infection between October 1st, 2010 and September 30th, 2015 were reviewed to determine the newly diagnosed TB infection. The personnel who were treated in fiscal year 2015 were interviewed about work-related issues, health status and the impact of TB.

**Findings:**

In five years, 109 personnel were diagnosed with new onset TB disease. The infection rates were 2.04, 1.97, 2.85, 2.53, and 1.35 per 1,000 persons in 2011, 2012, 2013, 2014, and 2015, respectively. The most prevalent type of TB infection was pulmonary TB. The infection rate in males was higher than in females. Pharmacists had the highest proportion of infected personnel. The second highest rate of infection was in support staff related to patient care. Twenty personnel were interviewed. Most of them worked in patient care units with central-type air-conditioning system without negative-pressure rooms for TB patients. Contracting TB had an impact on productivity at work, health (physically, mentally and socially) and incomes.

**Conclusions:**

Ramathibodi HCWs had higher rate of TB infection than the general Thai population, but the incidence was noted to be decreasing from 2013 to 2015. HCWs suffered from the impact of TB on their lives in multiple ways. Due to the adverse impact of TB on the health and welfare of its employees, hospital administration should apply effective preventive measures and develop a compensation system for HCWs infected with TB.

## Introduction

Work-related tuberculosis (TB) infections are common among healthcare workers (HCWs) [1,2,3,4,5]. In countries with a high TB burden, healthcare personnel are at higher risk than the general population [6]. According to Global TB Report 2017 by World Health Organization (WHO), Thailand is one of the high TB-burden countries [7] with the prevalence of TB 1.72 per 1,000 population in 2016 [8]. However, the incidence of new onset TB in HCWs is scarce and not current. Even in Ramathibodi Hospital, one of the leading university hospitals with almost 10,000 workers, only the incidence of tuberculin skin test (TST) conversion among newly recruited hospital personnel was investigated, which was 3.6% in 2009 [9]. A previous study conducted at another teaching hospital, King Chulalongkorn Memorial Hospital, during 1988–2002 reported that the overall TB incidence among HCWs was 1.88 per 1,000 person-years (py) [10].

TB disease is classified as pulmonary and extrapulmonary TB, based on the anatomical site of disease [11]. Patients who have TB involving the tracheobronchial tree or lung parenchyma, with or without extrapulmonary infections, are categorized as pulmonary TB. Extrapulmonary TB involves other organs, such as the intrathoracic lymph node, pleura, abdomen, etc. Although people with latent TB infection (LTBI) are at risk to progress to TB disease within a two-year period when they have recent TST conversions [12], LTBI is not included in this study.

The objectives of this study were to determine the five-year trend of TB infection rate among all HCWs at Ramathibodi Hospital, work-related factors, health problems, and the impact of TB on personnel whom were diagnosed with TB and treated with anti-TB drugs.

## Methods

A time series cross-sectional study was conducted at Ramathibodi Hospital, Mahidol University, Bangkok. It was a teaching hospital with 9,562 employees and approximately 1,000 inpatient beds. The study was divided into two phases. In the first phase, hospital numbers (HNs) of all HCWs in the hospital were obtained from the human resources department. Then the HNs were matched with the hospital database. The medical records of HCWs diagnosed with active TB or treated with anti-TB drugs between October 1st, 2010 and September 30th, 2015 (fiscal year 2011–2015) were reviewed by the principal investigator (PP) to determine the numbers of new cases of TB in each fiscal year, the inclusion criteria, treatments, and outcomes. Cases were considered having TB infection and were included in the study when they had: (1) positive sputum AFB, sputum culture for TB or imaging studies, or (2) completed courses of treatment for pulmonary or extrapulmonary TB. Cases were excluded when (1) diagnosed or had symptoms before working at the hospital, (2) resigned before the study period, (3) diagnosed as LTBI, or (4) had records with missing or unclear diagnoses. For the second phase, hospital workers diagnosed or treated in the fiscal year 2015 were interviewed for information about underlying health problems, history of BCG vaccinations, history of previous TB infections, history of contacting anyone with TB, work positions, job descriptions, workplaces, air-conditioning systems in workplace, infection control measures for TB patients, and the impact of having TB. For ethical reasons, underlying health problems were categorized as immunocompromised (e.g. diabetes mellitus, HIV infection), immunocompetent (e.g. hypertension, dyslipidemia), and none.

Quantitative data was analyzed by descriptive statistics using Microsoft excel 2013 and Stata statistical software version 14.0. Continuous data consistent with normal distribution was presented as means and standard deviations; otherwise, it was presented as median and interquartile range (IQR). Categorical data was presented as frequencies and percentages. Rates of new onset TB infection per fiscal year were calculated by dividing the number of new cases with mid-year worker population. Rate of TB infection in each department was divided by number of workers in that department. The pmeta command in Stata were used to estimate the average rates of TB infections and their confidence intervals. Content analysis was used on qualitative data.

The study was reviewed and approved by the Institutional Review Board of the studied hospital.

## Results

### Phase 1 Results

Between October 1st, 2010 and September 30^th^, 2015, TB (ICD-10 code A15–A19) was diagnosed in 8,521 patients. Of these, 245 were HCWs; however, 59 were given the wrong ICD codes, 20 were diagnosed before the study period or before working at the hospital, and 11 records were missing. Forty-six cases of LTBI were excluded. Finally, a total of 109 cases were included in the study.

Most of the HCWs with tuberculosis were female (71.6%). One-third were health management and other support staff (30.3%), followed by nurses (25.7%) and support staff related to patient care (23.9%). The median age was 32.9 years old, IQR 29.5–43.9 years old. One-half had worked for less than five years (50.5%), and most had no underlying health problems (64.2%) (Table 1).

**Table 1:** Characteristics of TB-infected hospital workers.

Characteristics	n	%	[95% CI]

Gender			
Female	78	71.6	[63.1, 80.0]
Male	31	28.4	[20.0, 36.9]
Age (years)			
Median 32.9, IQR 29.5–43.9			
20–29	31	28.4	[20.0, 36.9]
30–39	44	40.4	[31.2, 49.6]
40–49	18	16.5	[9.5, 23.5]
50 and over	16	14.7	[8.0, 21.3]
Years in service (years)			
Median 4.8, IQR 1.8–15.0			
Less than 5	55	50.5	[41.1, 59.8]
5–10	18	16.5	[9.5, 23.5]
More than 10	36	33.0	[24.2, 41.9]
Underlying disease			
Immunocompromised	22	20.2	[12.6, 27.7]
Immunocompetent	14	12.8	[6.6, 19.1]
None	70	64.2	[55.2, 73.2]
Unknown	3	2.8	[–0.3, 5.8]
Job position			
Physicians	18	16.5	[9.5, 23.5]
Nurses	28	25.7	[17.5, 33.9]
Pharmacist	4	3.7	[0.1, 7.2]
Support staff related to patient care	26	23.9	[15.9, 31.9]
Health management and other support	33	30.3	[21.6, 38.9]

When compared with total number of each gender, male staff had higher proportions of TB infection than female. The average TB infection rate in males was 3 per 1,000 persons (95% CI, 2–3); whereas, in females, it was 2 per 1,000 (95% CI, 1–3). The pharmacist position had the highest proportion of personnel with TB (Table 2). The second and third highest were support staff related and not related to patient care accordingly. Physicians and nurses were the fourth and fifth. Almost all of the infected physicians were residents in various training programs (72.2%).

**Table 2:** Pooled rate of TB infection among hospital workers categorized by job position.

Job position	per 1,000 employees	[95% CI]

Physicians	2	[1, 3]
Nurses	1	[1, 2]
Pharmacists	6	[0, 13]
Support staff related to patient care	3	[2, 4]
Health management and other support	2	[1, 3]

**Figure 1:**
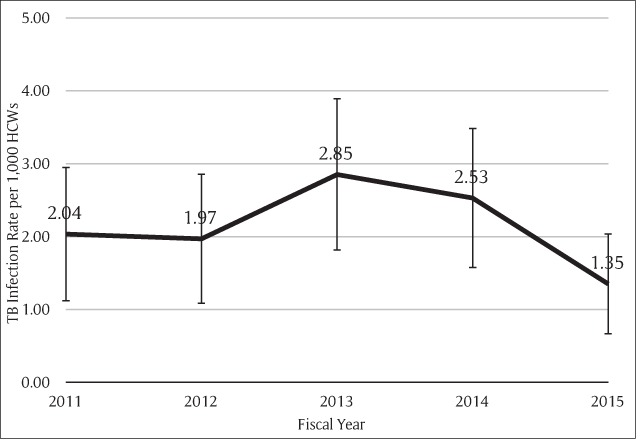
Rate of TB Infection among HCWs at Ramathibodi Hospital.

**Figure 2:**
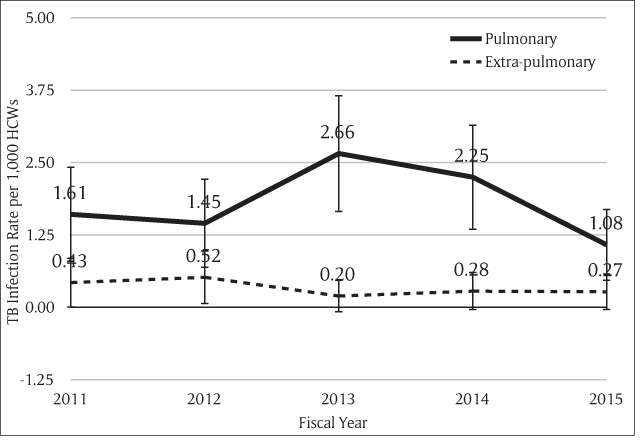
Types of TB Infection among HCWs at Ramathibodi Hospital.

The incidence of newly diagnosed TB infection among all HCWs were 2.04, 1.97, 2.85, 2.53, and 1.35 per 1,000 persons in fiscal year 2011, 2012, 2013, 2014, and 2015, respectively (Figure 1). The 5-year average rate of active TB disease was 2 per 1,000 persons. The majority of the cases in every year were pulmonary TB (Figure 2), 78% of total cases (Table 3). Most of the cases with pulmonary TB had their sputum sent for AFB stain (81.2%), but only 18.8% of them had positive results. Sputum cultures were also sent for 78.3%, and 44.4% of them had *Mycobacterium tuberculosis* growth. Among extrapulmonary TB cases, one had multidrug-resistant TB.

**Table 3:** Types of TB diseases.

Type	n	%	[95% CI]

Pulmonary TB	85	78.0	[70.2, 85.8]
Extrapulmonary TB	24	22.0	[14.2, 29.8]
Pleura	8	7.3	[2.4, 12.2]
Lymph node	7	6.4	[1.8, 11.0]
Peritoneum	1	0.9	[–0.9, 2.7]
Ileum	1	0.9	[–0.9, 2.7]

Standard drug regimen (2HRZE+4HR) was mostly given to cases with TB disease (60.6%). Overall, 83.5% of HCWs with TB completed their treatment and follow up, and 15.6% had recorded adverse effects from TB medications.

### Phase 2 Results

In fiscal year 2015, 39 HCWs were diagnosed with or treated for new onset TB. However, two had resigned from the hospital, nine refused to give interviews, seven were unreachable by phone, and one had symptoms before working at the hospital. Twenty HCWs with TB were interviewed between December 2015 and January 2016.

Seventeen of the interviewed personnel were female, with ages ranging from 23 to 55 years. Thirteen had received a BCG vaccine since birth, and one was treated for LTBI about eight years ago. Almost all of them worked in patient care units with a central air-conditioning system installed: eight in outpatient departments (OPDs), five in inpatient departments (IPDs), three in both OPDs and IPDs, and four in the emergency department (ED). The rests worked in back offices.

Two-thirds of the interviewees had pulmonary TB. More than half of them were asymptomatic and detected by routine health checkup. The rests had extrapulmonary TB.

Thirteen participants reported having contact with TB patients. Only six wore N95 masks for protection because others were unaware that those patients had active pulmonary TB. Twelve had other co-workers who were also diagnosed with TB. Airborne-infection isolation rooms, i.e. rooms with negative pressure ventilation, 12 air-changes per hour, and anterooms, were available in only three IPDs out of eight. These rooms were sometimes occupied by non-TB patients during the year of study. Moreover, almost all OPDs were not equipped with an airborne-infection isolation room.

Adverse effects of anti-TB drug were common. The most frequently observed were minor skin rashes, whereas the worst case that had drug reaction with eosinophilia and systemic symptoms (DRESS) was also seen.

In addition, infected HCWs suffered from negative impacts of TB on several aspects of their lives: (1) work, productivity and income, (2) family, and (3) social impact. First of all, hospital regulations required persons with pulmonary TB to take sick leave during the first two weeks of treatment. Therefore, they could not take overtime duties during the leave. Moreover, workers who had shift work reported feeling “too tired” to continue working on night shifts after day shifts while taking medications. As a result, they earned less income. Furthermore, their family members had to undergo TB investigations and one worker’s child was treated for LTBI. Finally, social impact was significant for many workers. Some felt isolated when they needed to stop working and stayed at home during the first two weeks. Although some did not have smear-positive pulmonary TB, they had to face the discrimination from their co-workers and friends. Some even violated the rule by not taking sick leave to avoid suspicions from their colleagues that they had TB.

## Discussion

The average rate of TB infection in HCWs in our study was higher than in general population [8] as observed in many studies [6,10,13]. Moreover, correlations between the numbers of TB patients in care, infection control measures, and rate of TB infection among HCWs were established [13]. When compared with the rate in King Chulalongkorn Memorial Hospital ten years ago, our TB incidence rate was slightly higher. The rate of tuberculosis infection decreased from 2.85 per 1,000 persons in fiscal year 2013 to 1.35 per 1,000 persons in fiscal year 2015. This could result from preventive measure implementations or under-diagnosis because the routine health checkup was skipped in 2015. The routine health checkup could detect many of HCWs with pulmonary TB, which is the majority of all TB infections in our workers.

Characteristics of HCWs with TB were somewhat similar to other studies. The median age was close to a study at a university hospital in Romania [14] and slightly less than a study in New York City [15]. Male employees had higher rate of infection than female, like in general Thai population [8]. It could be explained by the higher number of smokers among men than women, and tobacco smoking is one of the risk factors of TB infection [16]. Our pharmacists had the highest proportion of TB-infected workers. There was an outbreak in a pharmacy unit where the index case was a newly recruited worker who was diagnosed at pre-employment examination. Also, there were very small total numbers of employees in pharmacy positions compared to other job positions. Support staff related to patient care, such as laboratory workers, were the second highest. Laboratory staff in a study in Peru were also found to have higher rate of infection [17]. Among physicians, 72.2% of TB-infected physicians were residents. Similarly, a study in Mexico also found that physicians in training were at higher risk [18]. In our study, it could result from faculty physicians hardly participated in the health checks. Although some studies found that year in service has a positive correlation with TB infection [19], our study showed that most of the infected workers had worked for less than five years. The explanations could be that new employees were more likely to comply with hospital policy to have health checkup.

From the medical records, our workers’ TB treatment completion was 83.5%. The number was similar to the national TB profile, 79% [8]. However, we expected that the completion should be better, as HCWs had relatively fewer barriers than the general population.

Preventive measures could reduce TB infection in a healthcare setting as suggested by the US experience [20]. From the interview, we discovered that many of the hospital’s preventive policies were inadequately implemented. For engineering controls, there were not enough airborne-infection isolation rooms in OPDs and IPDs. For administrative controls, the communication systems about patients’ diagnoses were insufficient as some workers contacted TB patients without knowing the diagnosis. Therefore, they did not apply appropriate controls available, e.g. N95 respirators. Moreover, some workers avoided two-week sick leave, yet there were no consequences on those workers’ practices.

There were substantial negative impacts of TB among HCWs. Studies in general population provided similar themes [21,22,23,24], which were divided into physical, psychological, social, function, and financial aspects. We were surprised that there were discriminations and stigmas towards HCWs with TB. This could reflect inadequate TB education to HCWs due to assumption that they already knew. Although HCWs with TB were not confirmed that they were infected from work, compensations should be provided to alleviate the problems.

This study has several strengths. First of all, information of diagnoses and treatment were from the medical records, which were reviewed by a physician. The number of HCWs with TB was quite large. Data retrieved from the medical records would cover almost all of Ramathibodi HCWs because they usually received care at the hospital, as the costs could be reimbursed and it was more convenient. Moreover, it included all types of TB infection and reflected the trend from five years of data. In addition, the study consisted of qualitative data to help improve the understanding of the impacts. The interviews were conducted among personnel diagnosed and treated in 2015 to avoid recall problems.

A major limitation of our study was that HCWs who sought care elsewhere would not be included. Besides, ICD-10 miscoding as TB were identified; therefore, there could be some HCWs with TB coded as other diseases then not included.

These research results implied that the trend in HCWs with TB in our hospital is decreasing; however, HCWs’ average rate of TB infection is still higher than the general population. Therefore, the hospital should develop and enforce effective infection control, especially engineering control, and surveillance system to monitor the trend of TB infection in HCWs. TB education was necessary, even for HCWs, to help them understand proper personal prevention practices. We felt that there is also the need to reduce discrimination, as well as stigmatization among HCWs with TB. Moreover, methods to identify work-related TB infection should be considered. Whether or not the TB infection was work-related, HCWs suffered from substantial negative impacts; therefore, a compensation system should be made available for HCWs with TB.

## Conclusion

Ramathibodi HCWs had higher rate of TB infection than the general Thai population, but the incidence was noted to be decreasing from 2013 to 2015. HCWs suffered from the impact of TB on their lives in multiple ways. Due to the adverse impact of TB on the health and welfare of its employees, hospital administration should apply effective infection control strategies, implement a TB surveillance system, and develop a compensation system for HCWs infected with TB.
